# Circadian oscillations of cytosine modification in humans contribute to epigenetic variability, aging, and complex disease

**DOI:** 10.1186/s13059-018-1608-9

**Published:** 2019-01-03

**Authors:** Gabriel Oh, Karolis Koncevičius, Sasha Ebrahimi, Matthew Carlucci, Daniel Erik Groot, Akhil Nair, Aiping Zhang, Algimantas Kriščiūnas, Edward S. Oh, Viviane Labrie, Albert H. C. Wong, Juozas Gordevičius, Peixin Jia, Miki Susic, Art Petronis

**Affiliations:** 1 The Krembil Family Epigenetics Laboratory, The Campbell Family Mental Health Research Institute, Centre for Addiction and Mental Health, Toronto, Canada; 2 Institute of Biotechnology, Life Sciences Center, Vilnius University, Vilnius, Lithuania; 3 Center for Neurodegenerative Science, Van Andel Research Institute, Grand Rapids, MI USA

**Keywords:** Epigenetics, DNA modification, Methylation, Circadian, Differentiation, Aging, Disease, Schizophrenia, Leukemia, Diabetes

## Abstract

**Background:**

Maintenance of physiological circadian rhythm plays a crucial role in human health. Numerous studies have shown that disruption of circadian rhythm may increase risk for malignant, psychiatric, metabolic, and other diseases.

**Results:**

Extending our recent findings of oscillating cytosine modifications (osc-modCs) in mice, in this study, we show that osc-modCs are also prevalent in human neutrophils. Osc-modCs may play a role in gene regulation, can explain parts of intra- and inter-individual epigenetic variation, and are signatures of aging. Finally, we show that osc-modCs are linked to three complex diseases and provide a new interpretation of cross-sectional epigenome-wide association studies.

**Conclusions:**

Our findings suggest that loss of balance between cytosine methylation and demethylation during the circadian cycle can be a potential mechanism for complex disease. Additional experiments, however, are required to investigate the possible involvement of confounding effects, such as hidden cellular heterogeneity. Circadian rhythmicity, one of the key adaptations of life forms on Earth, may contribute to frailty later in life.

**Electronic supplementary material:**

The online version of this article (10.1186/s13059-018-1608-9) contains supplementary material, which is available to authorized users.

## Background

Circadian rhythmicity, an evolutionary adaptation to day and night cycles, influences a wide range of biological phenomena in virtually all life forms on Earth [1]. The circadian machinery helps organisms to coordinate metabolic and physiological processes, as well as adapt their behavioral activities, to the cyclically changing environment [2]. For mammals, environmental cues, like light and food, act as primary Zeitgebers (“time givers”) and play a key role in the synchronization of the organism’s internal biological rhythm with the day-night cycle [1]. The suprachiasmatic nucleus in the brain is the central pacemaker, but cell-autonomous circadian clocks in peripheral tissues can be maintained independently [3]. On a cellular level, the circadian molecular machinery is driven by a delayed negative feedback loop; the Clock and Arntl heterodimer complex activates genes encoding Per and Cry, which in turn suppress the heterodimer complex [4].

There is increasing evidence that circadian rhythm disturbances have adverse health effects. Impairment of oscillation mechanisms and sleeping patterns has been linked to various human morbidities, including cancer, psychiatric, and metabolic diseases [5]. For instance, circadian genes and their dysregulation were shown to be involved in tumors [6, 7]. Disturbed sleep and circadian dysregulation are an integral part of most mental disorders and may even play an etiological role [8]. Genome-wide association studies have also identified core circadian pathways as genetic risk factors in type II diabetes [9, 10]. Consistently with human findings, knocking out circadian genes in rodents resulted in a range of metabolic aberrations [11–13]. Despite clinical, epidemiological, and some molecular evidence that circadian dysfunction is related to complex diseases, the molecular mechanisms of these associations remain poorly understood.

**Fig. 1:**
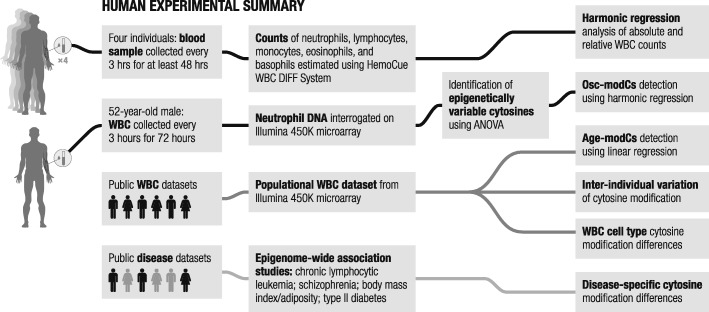
Experimental workflow summary. Cell count measurements were taken from four individuals across 48 h. Oscillating cytosines were identified in human neutrophil samples collected from a healthy male interrogated on the Infinium HumanMethylation450K BeadChip (Illumina). We utilized publicly available datasets to uncover the circadian effects in cytosine modification differences between white blood cell fractions [21], epigenetic variation and aging [31, 32], and disease-specific cytosine modification changes [32, 34–37]. Age-modC, age-correlated cytosine modifications; osc-modC, oscillating modified cytosines; WBC, white blood cells

Our group recently discovered evidence of circadian cytosine modification in mice [14]. We found that oscillating modified cytosines (osc-modCs) are prevalent in the mouse genome. Oscillating cytosines also exhibited age-dependent modification changes, and their oscillation amplitudes strongly correlated with the magnitude of the aging effect. In this study, we investigated osc-modCs in purified neutrophils collected from a healthy subject using the Illumina Infinium HumanMethylation450K BeadChip (Fig. 1). Unlike group-based circadian samples in animals, a single individual circadian dataset is not confounded by differences in external environment or DNA sequence variation, making it an ideal dataset to explore the effects of circadian rhythmicity on the epigenome. We show that osc-modCs can explain a part of both intra- and inter-individual epigenetic variation. Osc-modCs are overrepresented in the distal gene regulatory regions and are associated with epigenetic aging. Most importantly, osc-modCs are overrepresented in epigenome-wide association study (EWAS) hits for several complex diseases, suggesting the presence of a ubiquitous set of epigenetic disease risk factors that require extensive further investigation.

## Results

### Circadian oscillations of white blood cell fractions can simulate epigenetic oscillations

**Fig. 2:**
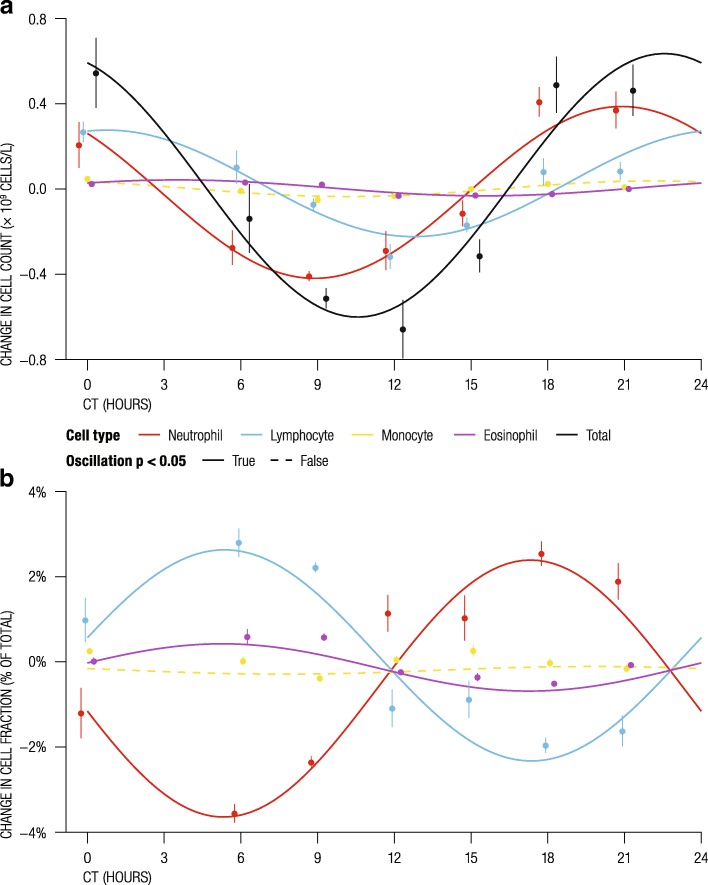
Circadian oscillations of absolute and relative cell counts in human white blood cell types. **a**, **b** Dynamics of **a** absolute count of four WBC types, and **b** proportion of each cell type relative to the total WBC count in four male subjects. Values are mean-centered by cell type, and solid line type indicates oscillation significance (*p* < 0.05). For each of the four subjects, data points with the same time of day were averaged. The lines represent harmonic regression fits, and whiskers represent the 95% confidence intervals of the between-subject mean. Data points are shifted slightly along the *x*-axis from their integer values to avoid whisker collisions. CT, circadian time

Blood samples (i.e., white blood cells (WBC)) are commonly used in molecular studies of human subjects due to their ease of access and relatively non-invasive collection procedure. Blood-based epigenomic analyses, however, can be confounded by WBC count differences across individuals and may generate false epigenetic effects [15]. Previous studies have shown that WBC counts oscillate in a circadian manner and the composition of cell types can change within an individual throughout the day [16]. We investigated WBC fractions collected every 3 h for at least 48 h from four male subjects and found that the total WBC count, as well as the number of different cell types in WBC, do indeed oscillate in a circadian manner (Fig. 2a; Additional file 1). Moreover, while absolute cell counts of neutrophils and lymphocytes oscillated in phase with the total blood count (Fig. 2a), their relative proportions were not uniform. For instance, lymphocytes were relatively enriched at around circadian time (CT) 6, while neutrophils were enriched at CT18 (Fig. 2b). This shows that failure to account for circadian cell count effects may simulate false epigenomic oscillations. Computational approaches [17] can be used to account for changes in cellular proportions but osc-modCs that correlate with cell counts may also be eliminated and result in a false negative outcome.

In order to avoid these confounders, we performed a circadian epigenomic analysis on a pure population of WBC. For our experiment, we opted to investigate neutrophils, which are the largest WBC fraction and exhibit substantial cytosine modification variability in the general population [18]. Some degree of neutrophil heterogeneity may exist [19], but it is currently unknown if the epigenomes of neutrophil subtypes differ significantly. Given that neutrophil subtypes have not been clearly defined, we used bulk neutrophils for our experiment.

### Mapping of modified cytosines reveals circadian patterns in human neutrophils

Blood samples were collected every 3 h over a 72-h period from a healthy 52-year-old male, who did not use any sleep-inducing medication, nor reported insomnia, hypersomnia, or other sleep disorders. We used magnetic bead-based antibody selection for separation of neutrophils and reached 98–99.5% (mean ± SD = 99.0 ± 0.56%) purity based on Houseman’s algorithm estimates of cell composition [17]. Cytosine modification profiles were interrogated using the Illumina HumanMethylation450 BeadChip. To reduce batch effects, every DNA sample was interrogated in technical duplicates. We selected cytosines whose biological modification signal significantly exceeded technical noise, which we refer to as epigenetically variable cytosines (EVCs; Additional file 2; see the “Methods” section for more detail).

**Fig. 3:**
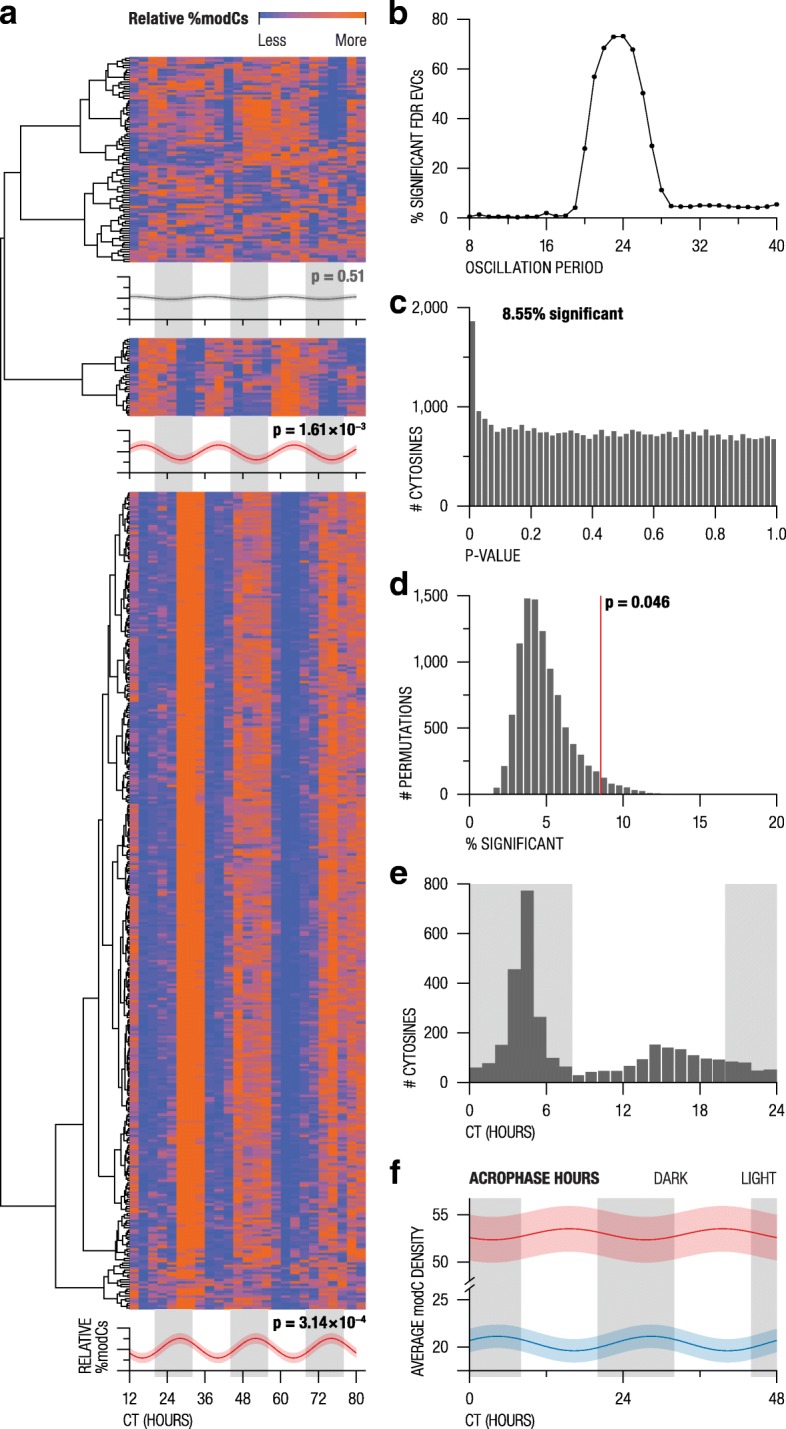
Oscillation profiles within epigenetically variable cytosines. **a** Heatmap of all FDR-significant EVCs with rows representing independent cytosines and columns representing samples ordered by CT. Missing CTs (CT31 and CT52) were replaced with the mean values of their two nearest neighbors. All cytosines were standardized using a *z*-score transformation. Hierarchical clustering with correlation distance grouped the cytosines into three clusters. Cosinor model fit on the averaged sample values is depicted below each cluster along with the cosinor *p* value. **b** Periodogram showing percentages of oscillating FDR EVCs using various oscillation periods. **c** Histogram of oscillation *p* values of nominally significant EVCs. **d** Percentage of oscillating nominally significant EVCs in 10,000 permutations of CT labels. The red line shows the observed percentage of oscillating cytosines in the unshuffled data. **e** Distribution of acrophases across significantly oscillating nominally significant EVCs. The gray shaded area indicates dark hours. **f** Average mesor values for osc-modCs peaking during light hours (red) and dark hours (blue). Shaded areas depict the 95% confidence interval for the mesor means. For illustration purposes, the mesor values were depicted using the average oscillation pattern within each group. CT, circadian time; modCs, modified cytosines; EVCs, epigenetically variable cytosines

Out of 485,512 interrogated cytosines, 466 (0.1%) were identified as EVCs after correction for multiple testing (false discovery rate (FDR) *q* < 0.05). A significant 24-h oscillation pattern (*p* < 0.05), estimated using the cosinor model [20], was detected in 73.18% of the FDR-significant EVCs (*p* = 8 × 10^−4^ after 10,000 permutations) (Fig. 3a). We found no evidence for the presence of other oscillation periods (Fig. 3b), suggesting that the 24-h period oscillations represent a predominant source of cytosine modification dynamics in neutrophils.

In order to capture more osc-modCs across the genome, we relaxed the EVC filtering threshold to nominal significance (ANOVA *p* < 0.05), which increased the subset to 38,410 (7.91%) cytosines. For every EVC, oscillation parameters, such as amplitude, acrophase (time of the oscillation peak), and mesor (rhythm adjusted mean), were estimated using a cosinor model [20] with a fixed 24-h period. We found that 8.55% of the EVCs (3238 of 38,410; permutation *p* = 0.045) showed significant oscillation (Fig. 3c, d), with a mean amplitude of 1.72% (range 0.09–8.32%). The majority of osc-modCs had acrophases between CT4 and CT6, with a smaller cluster positioned between CT15 and CT18 (Fig. 3e). These findings are in line with the results of our mouse study where oscillation acrophases had a bimodal distribution, roughly 12 h apart [14]. The bimodality of acrophases in both mouse and human tissues indicates that, at any given time, circadian modification effects are bi-directional; that is, some cytosines become methylated, while others are demethylated.

Light (CT8–20) and dark (CT20–8) hour acrophases also showed bimodality of the average modification density. Osc-modCs peaking during dark hours predominantly had low levels of modification (average mesor 20.3% [19.1–21.6%]), while osc-modCs with light acrophases were more heavily modified (average mesor 53.3% [50.9–55.7%]) (Fig. 3f). Similar to the mouse findings [14], the two patterns of human cell oscillations demonstrated cyclical divergence and convergence of cytosine modification densities, which we dubbed as epigenetic “apogee” (i.e., distance between the two sinusoidal curves reached their maximum) and “perigee” (i.e., distance between the two sinusoidal curves reached their minimum).

**Fig. 4:**
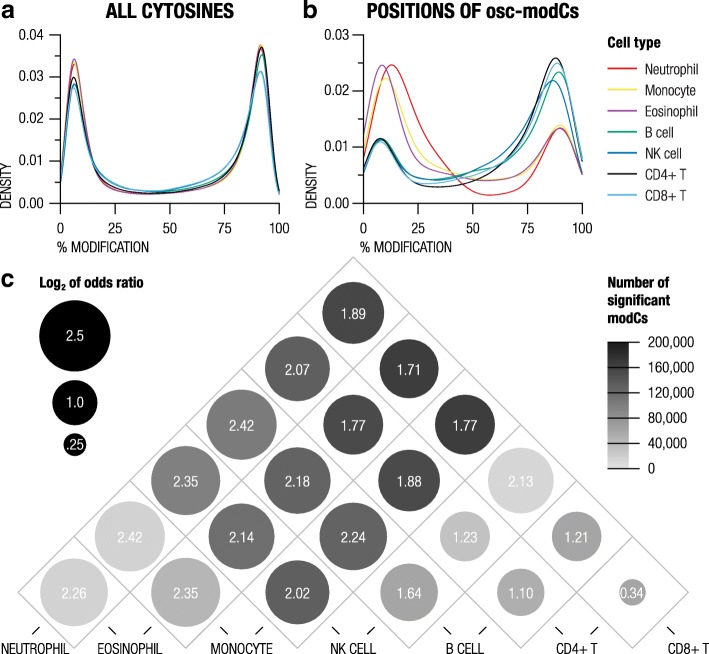
Association between oscillating cytosines and modification differences in cell lineages. **a**, **b** Cytosine modification densities of seven purified white blood cells within **a** all measured cytosines and **b** cytosines that were identified as oscillating in neutrophils. **c** Overlaps between neutrophil osc-modCs and differentially modified cytosine positions in various pairs of blood cell fractions. The size of the circles depicts log_2_ odds ratio of the overlap, and the shading represents the number of FDR-significant modification differences detected between pairwise comparisons of cell types. modC, modified cytosine

We examined cytosine modification densities of seven WBC types (myeloid lineage: neutrophil, monocytes, eosinophils; lymphoid lineage: B cells, NK cells, CD4+ T cells, and CD8+ T cells) from a public dataset composed of 6 unrelated blood donors [21]. Although all WBC types had similar overall cytosine modification profiles (Fig. 4a), at the counterpart positions to neutrophil osc-modCs, lymphoid cells had vastly higher modification densities compared to myeloid cells (Fig. 4b). Pairwise comparisons of the WBC types showed that differentially modified positions were associated with neutrophil osc-modC sites (Fig. 4c). Although the strongest overlaps were detected within myeloid lineage cells, neutrophil osc-modCs also overlapped with loci that were differentially modified within lymphoid lineage cells (e.g., B cells vs. CD4+ T cells). Assuming that our observations are not driven by an unknown neutrophil subtype heterogeneity, this finding suggests that osc-modCs may not be limited to neutrophils and that epigenomic oscillations may be involved in blood cell differentiation.

### Osc-modCs contribute to both intra- and inter-individual epigenetic variation

This neutrophil dataset from a single individual is not confounded by the effects of external environment and DNA variation, which allowed us to explore the contribution of osc-modCs to intra-individual epigenetic variability. As expected in the presence of true oscillations, we found that the proportion of intra-individual variance explained by osc-modCs increased with more stringent EVC selection threshold (Additional file 3). For instance, at ANOVA *p* < 0.05, 8.5% of EVCs were identified as osc-modCs and explained 8.5% of the variance (cosinor *p* = 3.1 × 10^−3^) reflected in the third principal component (PC). At FDR q < 0.05, however, 73.2% of the EVCs were found to be osc-modCs and explained 53.1% of the variance (cosinor *p* = 9.4 × 10^−4^) in the first PC. Traditionally, in the absence of a time dimension, EVCs would have been deemed stochastic. However, our data shows that the most dynamic parts of the epigenome over the duration of a day, within an individual, can be substantially attributed to osc-modCs.

**Fig. 5:**
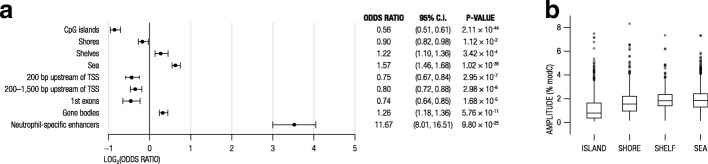
Relationships between osc-modCs and genomic elements. **a** Odds ratios of overlap between osc-modCs and various genomic elements estimated using a two-sided Fisher’s exact test. Full circles mark the log_2_ odds ratios, and extending bars represent 95% confidence intervals. **b** Box plots showing the distribution of osc-modC amplitudes in relation to genomic regions. modC, modified cytosine

Next, we examined the distribution of osc-modCs across various genomic elements and made three observations. First, sequences surrounding osc-modCs were enriched for canonical (CANNTG) and non-canonical (CANNNTG) E-box response element motifs (*e* value = 1.0 × 10^−23^–6.7 × 10^−60^) (Additional files 4 and 5), which play a key role in the regulation of circadian transcripts [22, 23]. We also identified enrichment of transcription factor motifs related to cellular differentiation and development (e.g., forkhead box (FOX), Fos-related, Jun-related, and Krüppel-related factors) [24–26], as well as immunity (e.g., interferon-regulatory factors) [27]. Secondly, oscillating cytosines were highly overrepresented in neutrophil-specific enhancer regions [28] (OR = 11.7 [8.0–16.5]; *p* = 9.80 × 10^−25^) (Fig. 5a). Lastly, osc-modCs were underrepresented at CpG islands and shores (0–2-kb region outside CpG island) but overrepresented in the shelves (2–4-kb region outside CpG island) and seas (regions farther than 4 kb from CpG island) (Fig. 5a). Contribution of osc-modCs to epigenetic variance was different across these regions. For instance, the average amplitude of oscillations was lower in CpG islands compared to the seas (average amplitude difference = 0.7%, *t* test *p* = 1.19 × 10^−44^) (Fig. 5b). Relatedly, oscillating cytosines were depleted within and near transcription starting sites but were enriched within gene bodies (Fig. 5a). Therefore, our data consistently indicates that osc-modCs may be important in regulating gene transcription.

Low cytosine modification variability in CpG islands, as well as increased variability in the more distal elements, has also been detected in cross-sectional studies of human epigenomes [29, 30]. To investigate the links between inter- and intra-individual variations, we used a publicly available list of cytosines exhibiting high degree of modification variability from a populational neutrophil dataset [18] and detected a strong overlap with osc-modCs (OR = 15.3 [12.3–19.0]; *p* = 2.52 × 10^−75^). Consistent with our osc-modC findings, the populational neutrophil sample exhibited depletion of epigenetic variability in CpG islands and proximal sites, while enrichment was observed in distal regions and enhancers [18]. Furthermore, motif enrichment analysis on sequences flanking populational neutrophil hyper-variable CpGs showed an enrichment for non-canonical E-box motifs (*e* value = 1.5 × 10^−22^) and Krüppel-related factors (*e* value = 1.2 × 10^−58^) (Additional files 6 and 7).

To further explore putative roles of osc-modCs in inter-individual variability, we re-analyzed two large whole blood datasets [31, 32], which were adjusted for white blood cell count differences, as well as known demographic, clinical, and technical covariates. The residual variation of modCs showed strong association with osc-modCs (logistic regression *p* = 1.80 × 10^−3^ and 4.36 × 10^−11^, for Hannum et al. and Hannon et al. datasets, respectively). Taken together, these findings suggest that, in addition to DNA sequence variation, non-shared environment, and stochasticity [33], osc-modCs may also contribute to the inter-individual variations of cytosine modifications.

### Osc-modCs are associated with aging

**Fig. 6:**
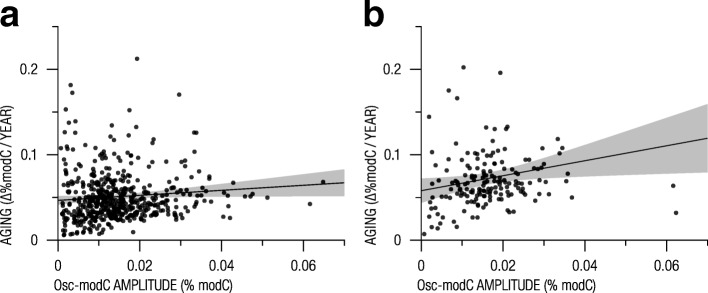
Association between osc-modCs and aging. **a**, **b** Scatterplot showing the relationship between aging magnitude and oscillation amplitude in two populational studies: **a** Hannum et al. (GSE40279) and **b** Hannon et al. (GSE80417). Black lines indicate fitted least squares regression lines with shaded gray area depicting 95% confidence intervals. Results produced using the subset of cytosines that exhibited both oscillating and aging effects. One outlier cytosine (Illumina probe ID “cg22454769”) was excluded from both figures for data visualization purposes. osc-modC, oscillating modified cytosine

In our previous mouse experiments, we detected that osc-modCs were associated with linear age-dependent cytosine modification changes [14]. In this study, we found that human neutrophil osc-modCs were also associated with age-correlated cytosine modification (age-modCs) in two whole blood datasets [31, 32] corrected for cell composition differences and various biological and technical covariates (OR = 1.53 [1.39–1.68]; *p* = 4.5 × 10^−17^ and OR = 1.39 [1.19–1.63]; *p* = 6.6 × 10^−5^, respectively). Like in the mouse liver and lung tissues, circadian amplitudes of human neutrophils correlated with the magnitude of epigenetic aging effects (Fig. 6a, b, Spearman’s rho = 0.11, *p* = 1.2 × 10^−2^ and rho = 0.23, *p* = 2.9 × 10^−3^). Finally, we replicated the observation that the time of osc-modC acrophase can predict the trend of epigenetic aging (but in opposite direction from mice that are nocturnal); cytosines with light hour acrophases were prone to accumulation of modified cytosines with age (OR = 1.88 [1.19–2.97]; *p* = 4.3 × 10^−3^ and OR = 2.47 [1.09–5.59]; *p* = 1.8 × 10^−2^)

### Osc-modCs are associated with complex diseases

Osc-modC’s involvement in cellular differentiation, epigenetic variation, and age-dependent epigenetic changes prompted us to investigate the roles of osc-modCs in complex diseases. We selected three different groups of diseases that represent major human pathological processes: malignancy (leukemia), neurodevelopmental dysfunction (schizophrenia), and metabolic dysregulation (obesity and type II diabetes).

**Fig. 7:**
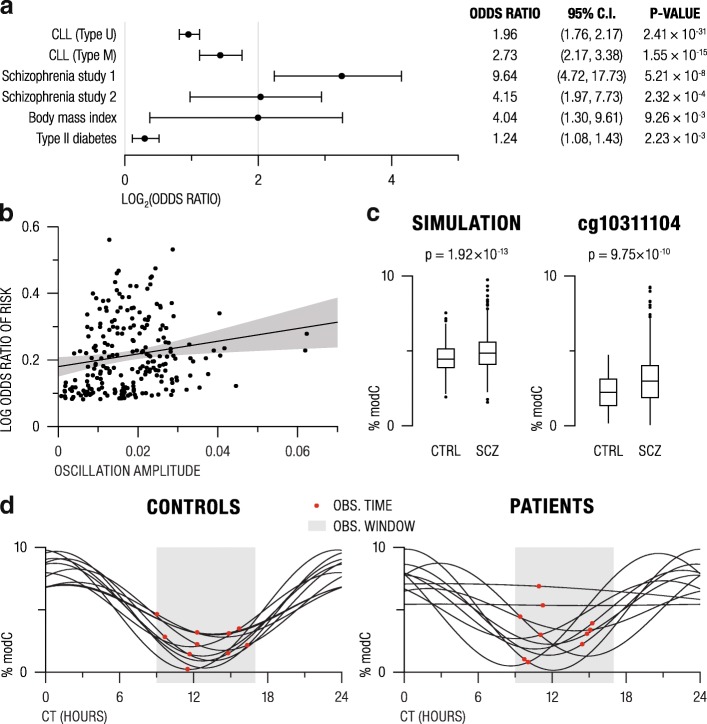
Association between disease and oscillating cytosine modifications. **a** Odds ratios of overlap between osc-modCs and differentially modified loci in various disease datasets estimated using Fisher’s exact test. Full circles mark log_2_ odds ratios, and whiskers represent 95% confidence intervals. **b** Scatterplot showing the association between the osc-modC amplitude and log-transformed odds ratio for type II diabetes risk. The black line indicates fitted least squares regression line with shaded gray area depicting 95% confidence interval. **c** Box plots of cytosine modification differences in simulated and a representative EWAS hit, cg10311104, from schizophrenia EWAS ([32]; Supplementary Fig. S5). Black dots represent outlier samples beyond the interquartile range. **d** Ten representative samples from osc-modC simulation in the “control” and the “patient” groups. Gray boxes represent the regular “office hours” (9 AM–5 PM) when samples are usually collected in a realistic clinical setting. Black curves represent the oscillation profiles for each sample, with red dots indicating a randomly selected sample collection time. CLL, chronic lymphocytic leukemia; C.I., confidence interval; modC, modified cytosine; CT, circadian time; CTRL, control; SCZ, schizophrenia; OBS, observation

We first investigated cytosine modification findings in chronic lymphocytic leukemia (CLL) [34], which is classified into unmutated (uCLL) and mutated (mCLL) based on the mutation status of the immunoglobulin heavy chain variable gene segment. We found that neutrophil osc-modCs were significantly overrepresented among differentially modified cytosines in B cells from both uCLL (OR = 1.96; *p* = 2.4 × 10^−31^) and mCLL (OR = 2.73; *p* = 1.6 × 10^−15^). Next, we analyzed three large blood-based EWAS, two of schizophrenia [32, 35] and one of body mass index (BMI) [36], and again detected significant overlaps between osc-modCs and EWAS hits from these three studies (Fig. 7a).

In order to uncover the direction of association between osc-modCs and disease, we utilized results from a prospective type II diabetes EWAS [37]. In this study, only seven cytosines were identified to predict type II diabetes at EWAS significance. Hence, we investigated cytosines with nominal *p* < 0.05 and found that osc-modCs were overrepresented in this group (OR = 1.24 [1.08–1.43]; *p* = 2.2 × 10^−3^). Interestingly, the magnitude of cytosine modification changes contributing to type II diabetes risk correlated with the osc-modC amplitudes (Spearman’s rho = 0.24, *p* = 2.4 × 10^−4^) (Fig. 7b), suggesting that osc-modCs can identify cytosine positions and magnitude of changes that are involved in type II diabetes. These findings are consistent with the causal interpretation; however, caution is necessary since type II diabetes is comorbid with obesity, and this association may reflect obesity-induced osc-modCs, rather than osc-modCs predisposing to diabetes.

It is important to note that all of the above comparisons were performed using data from mismatched cells (e.g., neutrophil osc-modC vs. B cells in leukemia) or non-primary targets of the disease (e.g., blood instead of neurons for schizophrenia). It is possible that associations between oscillating epigenetic factors and disease epimutations would be stronger if matching cell types were analyzed together.

### Disease EWAS hits may be cross-sectional “snapshots” of aberrant osc-modCs

Disease EWAS findings typically exhibit two properties. First, cytosine modification differences between affected individuals and controls, despite statistical significance, are very small. For example, the two large schizophrenia EWAS found mean absolute cytosine modification differences between cases and controls to be only 0.7% [35] and 1.3% [32]. Similarly, type II diabetes EWAS [37] found mean absolute differences in cytosine modification densities ranging from 0.5 to 1.1%. Second, patients quite often exhibit higher variability of cytosine modification compared to the controls [30, 38, 39]. In some studies (e.g., type I diabetes EWAS), enrichment of differentially variable cytosines in affected individuals compared to controls was the only statistically significant finding related to disease [29].

To investigate if these two groups of findings can be explained by the circadian epigenetic dysfunction in disease, we generated a simulated dataset with the following criteria: (1) Sample size was matched with previous schizophrenia EWAS [32]: 353 “patients” and 322 “controls”. (2) Amplitudes were randomly selected from a range of 3–10%. (3) We assumed that “controls” had conserved osc-modCs, while “patients” had disturbed circadian regulation. As such, oscillation period for “controls” was set to 24 h, while “patients” had 90% of samples with a 24 h period, 8% of samples with randomly selected periods ranging from 24 to 120 h, and 2% of non-oscillating samples. (4) Relatedly, acrophases for “controls” were randomly shifted within a 3-h interval (i.e., conserved), while “patient” acrophases were distributed across a wider interval of 9 h. (5) It was assumed that sample collection was performed within an 8-h time window, corresponding to regular working hours at a clinical setting (Fig. 7c, d).

The simulated dataset reproduced both of the common EWAS properties: a small but significant effect size (absolute mean difference = 0.85%; *t* test *p* = 1.92 × 10^−13^) and higher variance in “patients” compared to “controls” (variance ratio = 2.80; *F* test *p* = 4.8 × 10^−20^) (Fig. 7c). This suggests that single time recordings in the cross-sectional sampling traditionally used in EWAS may represent “snapshots” of aberrant circadian cytosine modifications and highlights the necessity of sample collection and analysis to be performed in a circadian-sensitive manner.

## Discussion

This neutrophil-based study identified several lines of converging evidence showing the importance of circadian oscillations of cytosine modification in humans. The discovery that cytosine modification is a part of the cellular circadian machinery is at odds with the traditional perception of static cytosine modifications in somatic differentiated cells, albeit with some gradual and unpredictable life-long “epigenetic drift” [40]. Our findings indicate that unexplained inter- and intra-individual variations of cytosine modification are not as random as once thought. Differential distribution of osc-modCs across genomic elements can be one of the reasons why regions outside of CpG islands exhibit higher variance in cytosine modification [41, 42]. Since epigenetic elements of higher variation are involved in tissue differentiation and malignant transformation (ibid.), osc-modCs may play a role in both processes. The observation of epigenetic “apogee” and “perigee” provide new mechanistic insights into carcinogenesis; if circadian epigenomic convergence is not fully compensated by divergence, the cytosine modification profile could acquire cancer-like features over a number of cycles, resulting in an extreme case of epigenetic “perigee.”

An overlap between osc-modCs from a single individual and positions of variable cytosine modification in the general population suggests that inter-individual epigenetic variability may be influenced by, at least to some extent, the circadian rhythm. Potential sites of population epigenetic variance may result from circadian differences among individuals, differences in the circadian time of sample collection, and (or) biological variation associated with osc-modCs (e.g., epigenetic aging). Our findings imply that differential epigenetic variation identified in several disease studies [29, 43–45] may also be associated with circadian epigenomic oscillations. If proven true, a direct link between circadian epigenomes and inter-individual epigenomic variation would provide a mechanistic basis for parts of ~ 80% of populational variation that is assumed to be of unexplained environmental origin [33].

While we purified neutrophils to eliminate between blood cell type heterogeneity, the study can still be confounded by circadian replenishing of the neutrophil subtypes [46]. Although neutrophils exhibit variation in density of surface antigens during maturation (e.g., CD62L) [46], current evidence suggests that cytosine modification profiles across different stages of development show no discernable differences [47]. In addition, the neutrophil study used for our intra- vs. inter-individual comparison [18] tested for the expression of several surface antigens, a proxy marker for cell type heterogeneity, and excluded neutrophil subpopulations as the main determinant of inter-individual cytosine modification variability. Nevertheless, neutrophil subtypes have not been clearly characterized, and interpretation of our findings cannot be completely transparent. The fact that WBC differentiating cytosines were significantly enriched for osc-modCs may imply hidden cellular heterogeneity. On the other hand, this may also indicate that osc-modCs are linked to cell differentiation and development. We believe that even if some cytosines were to be involved in daily dynamics of neutrophil subtypes, and therefore simulate oscillations, it is unlikely that they can fully account for the findings described in this study. Given the large number of EWAS that showed an association with our osc-modCs, where all studies were performed independently from each other, all datasets would have to be consistently confounded by inefficiently corrected heterogeneity. However, we acknowledge that our findings may be confounded by hidden heterogeneities and that our biological interpretation may change as new WBC subtypes are discovered. Moreover, even if our findings are the result of some yet unknown hidden neutrophil subtype heterogeneity, our biological interpretation may be incorrect but it does not diminish its property as an epigenetic marker of disease. Relatedly, since our subjects were exposed to normal lighting and eating habits during the experiments (i.e., external entrainment cues), it is also difficult to parse out the relationship between osc-modCs and intrinsic circadian rhythm. In short, there are many hurdles that impede our ability to fully interpret our findings, but future environment-controlled experiments involving large-scale molecular characterizations of neutrophils at a single-cell resolution may help resolve these question.

Independent from interpretation uncertainties, a shift from a “static and stochastic” cross-sectional studies to “cyclic and deterministic” circadian strategies can change our understanding of the molecular and cellular basis of common disease. Circadian strategies are based on multiple samples (WBC, adipocytes, fibroblasts, cultivated cells) collected over a 24-h period (or longer) to identify individual-specific profiles. Although the cause-and-effect relationship between disturbed circadian cycles and complex disease still needs to be established, the circadian interpretation of disease origin is simple and intuitive; daily circadian reprogramming is likely to be prone to errors and imperfectly maintained circadian aberrations in epigenomes (and transcriptomes, metabolomes, or cell subtypes) gradually convert into disease risk factors.

Circadian molecular and cellular studies may identify individual-specific disease features that could open new opportunities for precision medicine, and offer a customized approach for predicting disease risks and prognosis to facilitate early and efficient interventions [48]. Such approaches would integrate circadian biomarkers with clinical data to develop a more accurate molecular disease taxonomy to improve diagnostic specificity and treatment efficacy [49]. Furthermore, since circadian parameters can be modified by diet, lifestyle, and medications [50], we predict that preventative interventions aimed at rectifying circadian aberrations may be a viable approach to reduce the risk of a disease or delay its age of onset.

## Conclusions

Circadian oscillations of cytosine modification are implicated in the epigenomic trajectories of aging and common diseases, suggesting that evolutionary adaptive processes can mediate an organism’s frailty in later parts of its life. Future studies should focus on improving the biological interpretability by resolving confounders that we were unable to address in this study, such as potential neutrophil subtype heterogeneity and external entrainment cues on the intrinsic circadian rhythm (e.g., diet and light). However, in addition to the existing recommendations for population epigenomic studies [15], subjects and sample collection timing should be, at a minimum, matched for their circadian phase, as a systematic shift in collection times could result in mean modification differences that are conflated with disease-related dysregulation. Prospective studies are warranted for uncovering the direction of the association between the circadian epigenome and disease.

## Methods

### Sample collection and preparation

In order to measure white blood cell oscillations, cell count measurements were gathered using a point-of-care machine, the HemoCue WBC DIFF System (HemoCue, Sweden), from four male subjects for a minimum of 48 h (every 3 h starting at CT9 with the exclusion of the 3 AM collection). Each measurement was repeated three consecutive times by the subject in their homes following the manufacturer’s recommendations (manufacturer’s protocols were used for all other kits, unless stated otherwise). This machine measures relative and absolute levels of six different WBC fractions (neutrophils, lymphocytes, monocytes, eosinophils, basophils, and total WBC count) using 10 μL of blood in pre-stained microcuvettes.

For neutrophil isolation, a total of 20 venous peripheral blood samples were collected every 3 h for 72 h, starting at circadian time 13 (CT13), with one missing time point at CT52 (where CT13 corresponds to 1 PM in local time). Eight milliliters of blood was collected in EDTA Vacutainer tubes at each collection time from a 52-year-old Caucasian male. The subject typically sleeps from 12 AM to 8 AM and did not report significant changes to his sleeping pattern during the experiment.

Neutrophils were isolated immediately from the whole blood by immunomagnetic negative selection with an EasySep™ Direct Human Neutrophil Isolation Kit (STEMCELL Technologies, BC, Canada). This negative selection for neutrophils was repeated three times, and the cells were washed with phosphate-buffered saline, pelleted, and snap-frozen in liquid nitrogen. The neutrophils were stored at − 80 °C before DNA extraction. DNA extraction was performed with NucleoSpin® Blood XL (Macherey-Nagel) kit just prior to downstream experiments.

### Oscillating WBC fractions

Basophils were removed from the analysis due to their low count. For each time point, the mean of triplicate measurements for an individual, as well as the mean of all four individuals was calculated. These values were then fit using the cosinor regression model as described below.

### Bisulfite conversion and microarray experiment

A total of 750 ng of genomic DNA was bisulfite-converted using an EZ DNA Methylation™ Kit (Zymo) according to the manufacturer’s protocol for the HumanMethylation450 BeadChip (Illumina, CA, USA), with the following modifications suggested by the manufacturer for a more stringent conversion: 7.5 μL of M-dilution buffer was used for the reaction, which was incubated at 42 °C for 30 min prior to addition of the CT-Conversion Reagent. A total of 185 μL of the M-dilution buffer was used in the preparation of the CT-Conversion Reagent, and only 97.5 μL of the reagent was added per reaction.

HumanMethylation450 BeadChip assays were performed in duplicates using 500 ng of the bisulfite-converted genomic DNA at The Centre for Applied Genomics (Toronto).

### Pre-processing of the purified human neutrophil data

Raw data were processed using the “minfi” package [51]. Quality control using the control probes showed no notable aberrations. Normalization was performed using “noob” background correction [52] followed by Functional normalization [53], and signals from the methylated (modified) and unmethylated (unmodified) channels were combined to obtain the beta values. Cell count estimates were measured using Houseman’s algorithm implemented in the Bioconductor package “minfi” [51]. In order to reduce the influence of position effects, signal intensities were mean-centered by subtracting the Sentrix-specific mean beta values from each sample.

To identify outliers, all samples were internally correlated and samples with an average inter-sample correlation value more than two standard deviations below the mean were removed as outliers. This procedure identified a single sample at CT31 as an outlier, and both of its technical replicates were excluded from further analysis.

### Detection of epigenetically variable cytosines (EVCs)

Technically consistent and epigenetically variable cytosines (EVCs) were identified by comparing their technical and biological variation using a one-way ANOVA between the biological samples. In all subsequent analyses, only the subset of significant EVCs (*p* < 0.05) were considered for possible oscillation effects. In some cases, where specified, a more stringent threshold of FDR-significant EVCs (FDR *q* < 0.05) was used instead. Following this step, each biological replicate was averaged using the median of its technical replicates.

### Detection of oscillating modified cytosines (osc-modCs)

A cosinor model [20] was used to identify circadian oscillations. The period was fixed to 24 h, and the phase, mesor, and amplitude were modeled as a linear combination of sine and cosine terms as follows:

$$ y={b}_0+{b}_1\cdot \sin \left(2\pi \cdot CT/24\right)+{b}_2\cdot \cos \left(2\pi \cdot CT/24\right)+\varepsilon $$

where *y* is the observed modification level, *b*_*i*_ are regression coefficients, *CT* is the time of observation, and *ε* is the error term. *p* values were obtained by comparing this model to the null intercept-only model using an *F* test. EVCs with cosinor *p* < 0.05 were identified as osc-modCs.

To determine whether the observed proportion of oscillating cytosines was higher than expected by chance, 10,000 permutations were performed by shuffling CT labels, and the proportion of oscillating cytosines was calculated for each permutation. The permutation *p* value was derived as a fraction of permutations that had higher number of oscillating cytosines compared to the observed proportion in the unshuffled data.

Principal component analysis was used to quantify the amount of variability explained by oscillations within EVCs. Principal components were calculated via singular value decomposition of the mean-centered data matrix. The resulting scores of four main principal components were inspected for oscillations by fitting the cosinor model as described above.

### Osc-modC position profiles in white blood cell fraction

Public human white blood cell dataset [21] (data available in the BioConductor’s “FlowSorted.Blood.450 k” package [54]) was used to detect modification differences between distinct cell types. The dataset was normalized using subset quantile within-array normalization (SWAN [55]), and modification differences between all pairwise combination of seven WBC types (myeloid lineage: neutrophils, monocytes, eosinophils; lymphoid lineage: B cells, NK cells, CD4+ T cells, and CD8+ T cells) were estimated using a paired *t* test. For each pairwise comparison, only cytosines with FDR *q* < 0.05 were identified as differentially modified.

### Motif analysis

Sequence motifs were examined at the oscillating cytosine position ± 100 bp. Overlapping 200-bp regions (i.e., redundant sequences) were merged into one sequence. MEME suite 4.10.2 [56] was used to identify overrepresented sequences using the following parameters: -dna, -mod anr, -maxsites 1000, -nmotifs 10, -evt 1e-10, -revcomp, -maxsize 10000000. TOMTOM [57] from the MEME suite was used to identify enriched transcription factor motifs using the JASPAR 2018 CORE position frequency matrix (non-redundant) database for vertebrates [58] as a reference and using default parameters.

### Genomic element analysis

Positions of CpG islands, shores, shelves, transcription starting sites, first exons, and gene bodies were defined according to Illumina HumanMethylation450 array Manifest file v1.2 (GEO accession: GPL13534). Genomic positions of neutrophil-specific enhancers were taken from FANTOM5 human enhancer database [28]. Associations between osc-modCs and various genomic elements were estimated using a two-sided Fisher’s exact test.

### Identifying inter-individual epigenetic variation

Cytosines with hyper-variable modification in neutrophils were obtained from [18]. Association between hyper-variable cytosines and osc-modCs was estimated using a two-sided Fisher’s exact test.

We also utilized two whole blood public datasets (GEO accession: GSE40279 [31] and GSE80417 [32]). Beta values were quantile normalized, sex chromosome probes were removed, and principal components were calculated using 10,000 most variable cytosines. Samples deviating by more than two standard deviations from the mean on any of the first three principal component scores were identified as outliers and removed from further analysis. Final sample sizes used for analysis were *n* = 580 and *n* = 304 (healthy controls only, age < 100), respectively.

Stochastic variation of cytosine modification was estimated by measuring standard deviation on residuals after regressing out cell count estimates (CD8+ T cell, CD4+ T cell, CD8pCD28nCD45RAn memory and effector T cell, NK cell, B cell, monocyte, granulocyte, and plasmablast), technical variates (Sentrix ID and Sentrix row), and clinical information (age, sex, and smoking score) from the data. Cytosines positioned close to known SNPs (defined by Illumina HumanMethylation450 array Manifest file v1.2 (GEO accession: GPL13534)) and overlapping known methylation quantitative trait loci (list for middle age mQTLs obtained from [59]) were discarded. White blood cell count estimates were obtained using a DNA methylation age calculator [60]. Association of osc-modC and variability was modeled using a logistic regression with oscillation status as response variable and estimated stochastic variation as an independent variable.

### Identifying age-dependent modification changes

Age-dependent cytosine modifications were identified in the abovementioned datasets by performing an *F* test between a null linear model and a model with additional age covariate. The list of null model covariates included cell count estimates (CD8+ T cell, CD4+ T cell, CD8pCD28nCD45RAn memory and effector T cell, NK cell, B cell, monocyte, granulocyte, and plasmablast), Sentrix ID, Sentrix row, sex, and smoking score. Cytosines whose modification showed a significant (FDR *q* < 0.05) association with age were called age-correlated cytosines (age-modC), and the sign of age-related beta coefficient of the fitted linear model was used to determine the direction of change. White blood cell count estimates were obtained using a DNA methylation age calculator [60]. Associations between osc-modCs and aging were estimated using two-sided Fisher’s exact test.

### Identifying overlap between osc-modCs and EWAS significant cytosines

Probe IDs for chronic lymphocytic leukemia were obtained from Supplementary tables 6 (type U) and 7 (type M) [34]. The schizophrenia significant probes were obtained from Supplementary table 8 [32] and Supplementary table 3 [35]. The BMI-associated cytosine modification changes were obtained from Supplementary table 23 [36]. *p* values of cytosine association with type II diabetes were obtained from the authors of the publication [37]. Associations between osc-modCs and disease-related cytosines were estimated using a two-sided Fisher’s exact test.

### Programming language

All computational analyses were performed using R v3 [61] unless specified otherwise.

## Additional files

Additional file 1: Oscillation parameters of white blood cell fractions. Counts, fractions, and oscillation *p* values of 5 WBC cell types averaged across 4 individuals. (XLSX 9 kb)

Additional file 2: Oscillation parameters for all interrogated CpG positions. EVC *p* value, cosinor *p* value, mesor, acrophase, amplitude, and genomic information for all interrogated cytosine positions. (CSV 40045 kb)

Additional file 3: Proportion of variance explained by oscillations at various EVCs thresholds. Number of total EVCs, percent of oscillating EVCs, and summary of oscillating principal component scores at different EVC selection thresholds. (XLSX 9 kb)

Additional file 4: MEME output for oscillating cytosines in neutrophil. Motif enrichment logos and characteristics for the sequences within 100 bp of osc-modCs. Generated using the MEME software tool. (HTML 2357 kb)

Additional file 5: TOMTOM output for oscillating cytosines in neutrophil. Motifs detected from sequences flanking osc-modCs queried against a database of known vertebrate transcription factor sequence motifs. Significant matches are indicated beside each query. Generated using the TOMTOM software tool. (HTML 285 kb)

Additional file 6: MEME output for hyper-variable cytosines from populational neutrophil data. Motif enrichment logos and characteristics for the sequences within 100 bp of hyper-variable cytosines from populational neutrophil data. Generated using the MEME software tool. (HTML 949 kb)

Additional file 7: TOMTOM output for hyper-variable cytosines from populational neutrophil data. Motifs detected from sequences flanking hyper-variable cytosines in populational neutrophil data queried against a database of known vertebrate transcription factor sequence motifs. Significant matches are indicated beside each query. Generated using the TOMTOM software tool. (HTML 110 kb)

## Abbreviations

age-modC: Age-correlated modified cytosine
CT: Circadian time
EVC: Epigenetically variable cytosine
EWAS: Epigenome-wide association study
FDR: False discovery rate
osc-modC: Oscillating modified cytosine
